# First and second morning spot urine protein measurements for the assessment of proteinuria: a diagnostic accuracy study in kidney transplant recipients

**DOI:** 10.1186/s12882-021-02406-x

**Published:** 2021-05-22

**Authors:** Maja Mrevlje, Manca Oblak, Gregor Mlinšek, Jelka Lindič, Jadranka-Buturović-Ponikvar, Miha Arnol

**Affiliations:** 1 Department of Nephrology, Centre for Kidney Transplantation, University Medical Centre Ljubljana, Zaloska 7, 1000 Ljubljana, Slovenia; 2 Department of internal medicine, General Hospital Izola, Izola, Slovenia; 3 Faculty of Medicine, University of Ljubljana, Ljubljana, Slovenia

**Keywords:** Accuracy, Kidney transplantation, Protein-to-creatinine ratio, Proteinuria

## Abstract

**Background:**

Quantification of proteinuria in kidney transplant recipients is important for diagnostic and prognostic purposes. Apart from correlation tests, there have been few evaluations of spot urine protein measurements in kidney transplantation.

**Methods:**

In this cross-sectional study involving 151 transplanted patients, we investigated measures of agreement (bias and accuracy) between the estimated protein excretion rate (ePER), determined from the protein-to-creatinine ratio in the first and second morning urine, and 24-h proteinuria and studied their performance at different levels of proteinuria. Measures of agreement were reanalyzed in relation to allograft histology in 76 patients with kidney biopsies performed for cause before enrolment in the study.

**Results:**

For ePER in the first morning urine, percent bias ranged from 1 to 28% and accuracy (within 30% of 24-h collection) ranged from 56 to 73%. For the second morning urine, percent bias ranged from 2 to 11%, and accuracy ranged from 71 to 78%. The accuracy of ePER (within 30%) in first and second morning urine progressively increased from 56 and 71% for low-grade proteinuria (150–299 mg/day) to 60 and 74% for moderate proteinuria (300–999 mg/day), and to 73 and 78% for high-grade proteinuria (≥1000 mg/day). Measures of agreement were similar across histologic phenotypes of allograft injury.

**Conclusions:**

The ability of ePER to accurately predict 24-h proteinuria in kidney transplant recipients is modest. However, accuracy improves with an increase in proteinuria. Given the similar accuracy of ePER measurements in first and second morning urine, second morning urine can be used to monitor protein excretion.

**Supplementary Information:**

The online version contains supplementary material available at 10.1186/s12882-021-02406-x.

## Background

In kidney transplant recipients proteinuria is an independent indicator of kidney injury and predicts chronic kidney disease (CKD) progression and allograft loss [1–3]. The gold standard for proteinuria measurement is collection of 24-h urine samples. However, these collections are cumbersome for patients if they need to be collected frequently and, therefore, prone to under and over collection [4]. For everyday clinical practice, it is easier to estimate proteinuria by calculating protein-to-creatinine ratio (PCR) using spot urine samples [5, 6]. Previous studies have primarily examined the predictive value of PCR in the first morning sample or random spot sample of urine in patients with CKD of the native kidneys [7]. However, the validity of spot urine protein measurements in the kidney transplant recipients remains unclear. Studies on diagnostic accuracy of PCR in transplanted patients published to date mainly stated sensitivity and specificity and reported on PCR having excellent correlation with 24-h proteinuria [8]. Yet none of these measures give accurate information about the quantitative accuracy of the test to a clinician trying to determine how much proteinuria is truly present.

Etiology of proteinuria is different in kidney allografts than in native kidneys, and different levels of proteinuria in each result from different pathological mechanisms, as well as provide different information on graft and patient survival [9]. Transplant-specific diagnoses rather than native kidney diseases have been more commonly found on biopsies performed for proteinuria [10]. Low-grade proteinuria consists mostly of non-albumin proteins, whereas high-grade proteinuria consists mostly of albumin; pathohistological studies in transplant recipients reported mostly interstitial fibrosis and tubular atrophy in those with low grade proteinuria, and glomerular disease in the majority of allograft biopsies with high-grade proteinuria [3, 11]. Especially in patients with different levels of proteinuria and higher proportion of proteins of non-albumin origin, the predictive value of PCR in spot urine collections for assessing 24-h proteinuria remains unclear.

Only one study to date evaluated absolute agreement (i.e., bias, precision and accuracy) of PCR measurements and 24-h proteinuria in kidney transplant recipients [12]. Unfortunately, random spot urine PCR that were used as a representation of the 24-h urine collection show poor agreement with 24-h proteinuria [13]. Therefore, in our study we aimed to better clarify measures of agreement between estimated protein excretion rate (ePER) as determined from PCR in the first and second morning spot urine collections and 24-h proteinuria, and also to study their performance at different levels of proteinuria. Furthermore, we were interested in the measures of agreement of spot urine protein measurements in relation to different histologic phenotypes of allograft injury. Finally, we investigated excretion of different proteins (total protein, albumin, and tubular protein α-1 microglobulin) in the first and second morning spot urine collections and their relationship to 24-h proteinuria and allograft histology.

## Methods

### Study design

We performed an investigator-initiated, observational, cross-sectional study of adult deceased donor kidney transplant recipients that completed the ‘Paricalcitol versus placebo for reduction of proteinuria in kidney transplant recipients: a double-blind, randomized controlled trial’ (ClinicalTrials.gov, number NCT01436747) [14]. Briefly, between July of 2012 and October of 2014 the Paricalcitol trial recruited a national cohort of adult kidney transplant recipients with stages 1–4 chronic kidney disease (CKD) and residual proteinuria more than 3 months after transplant. Inclusion criteria were urinary PCR ≥20 mg/mmol despite optimization of the single-agent renin-angiotensin-aldosterone system blockade and an estimated glomerular filtration rate (eGFR) ≥15 mL/min/1.73m^2^. The study included a 12-week screening phase followed by a 24-week randomized treatment period and an 8-week follow-up after treatment withdrawal [14].

### Study participants

This follow-up diagnostic accuracy study included all study participants who were at least 3 months after Paricalctiol trial completion and had stable allograft function (serum creatinine variation < 20% from baseline during the previous 3 months) with an eGFR ≥15 mL/min/1.73m^2^, and residual 24-h urine protein excretion ≥150 mg/day/1.73m^2^. Patients having documented fever, urinary tract infection, indwelling urinary catheter, uncontrolled hypertension (blood pressure ≥ 160/100 mmHg), active malignancy, and pregnancy or breastfeeding were not candidates for the study. All patients provided written informed consent. The study protocol was approved by the National Medical Ethics Committee.

### Measurements

All patients who met the study inclusion criteria were subjected to spot and 24-h urine protein excretion analyses. One day before the study visit patients were instructed to collect and refrigerate (at 4–6 °C) midstream first morning void urine specimen and to begin the 24-h collection immediately after completion of the first morning void. The participants then collected all urine for 24 h, including the first morning void at the end of the 24-h period.

The next morning, after finishing the 24-h urine collection, the participants were asked to bring the first morning and 24-h urine specimens to the transplant clinic when a midstream second morning urine specimen was obtained. Urine collection procedure was repeated in patients who under or over collected the 24-h urine (creatinine excretion < or > 2 standard deviations [SD] of estimated creatinine excretion) [15]. First and second morning void urine collections were analyzed for protein, albumin, α-1 microglobulin and creatinine, and 24-h urine samples were analyzed for total protein and creatinine. Baseline demographics, clinical characteristics, vital parameters (blood pressure, pulse rate, body weight and height), medication use, and blood chemistry were also assessed on the day the 24-h urine collection was completed. Certified local laboratories were utilized to process and provide results for all laboratory tests. Further details on the study measurements are described in the supplementary documentation (Supplement file 1).

### Statistical analyses

The primary aim of the analysis was to assess the performance of PCR in first and second morning spot urine collections for estimating 24-h proteinuria. 24-h proteinuria was corrected for standard body surface area by multiplying the measured value by 1.73 and dividing it by the patients’ body surface area. Estimation of 24-h protein excretion rate (ePER, mg/day/1.73m^2^) was obtained by multiplying PCR and estimated creatinine excretion rate [12, 16]. Percent bias, precision, and accuracy were calculated for the following values of 24-h proteinuria: 150 to 299 (mild proteinuria), 300 to 999 (moderate proteinuria), and 1000 or more mg/day/1.73m^2^ (high-grade proteinuria) [17, 18]. Bias was defined as the mean difference between the measured value (24-h proteinuria) and the estimated value (ePER). Percent bias was defined as (bias per 24-h urine protein excretion) × 100 [19]. Precision was defined as the SD of the difference between measured and estimated value [19]. Accuracy was defined as the percentage of estimated values within 15, 30, and 50% of measured value [19]. Data were presented as mean (95% confidence interval) and number (percentage) for nominal data. *P* values for differences between the first and second morning urine samples were assessed with the dependent sample Wilcoxon signed rank test for continuous data and the chi-squared test for nominal data. The correlation between estimated and measured 24-h urine protein excretion was measured by Pearson’s correlation coefficients, and the degree of agreement by Bland-Altman analysis. Receiver operator characteristic (ROC) curves were used to obtain the best sensitivity and specificity of ePER in first and second morning urine collections at different cutoff levels of 24-h proteinuria.

To investigate the performance of ePER determined from PCR in the first and second morning urine in different histologic phenotypes of allograft injury, we reanalyzed data restricted to study patients with information on histologic diagnoses of allograft injury from the Paricalcitol trial [14]. There were 76 patients available for this analysis. All biopsy specimens were evaluated according to the Banff criteria for histologic lesions [20, 21]. *P* values for differences in spot urine protein, albumin, and α-1 microglobulin excretion between different histologic phenotypes were assessed with the Kruskal–Wallis test for non–normally distributed data.

A two-sided *P* value < 0.05 was used as the criterion for statistical significance. All analyses were performed using the SPSS statistical software (IBM SPSS statistics, version 21.0, Armonk, NY, USA).

## Results

### Study population

**Fig. 1:**
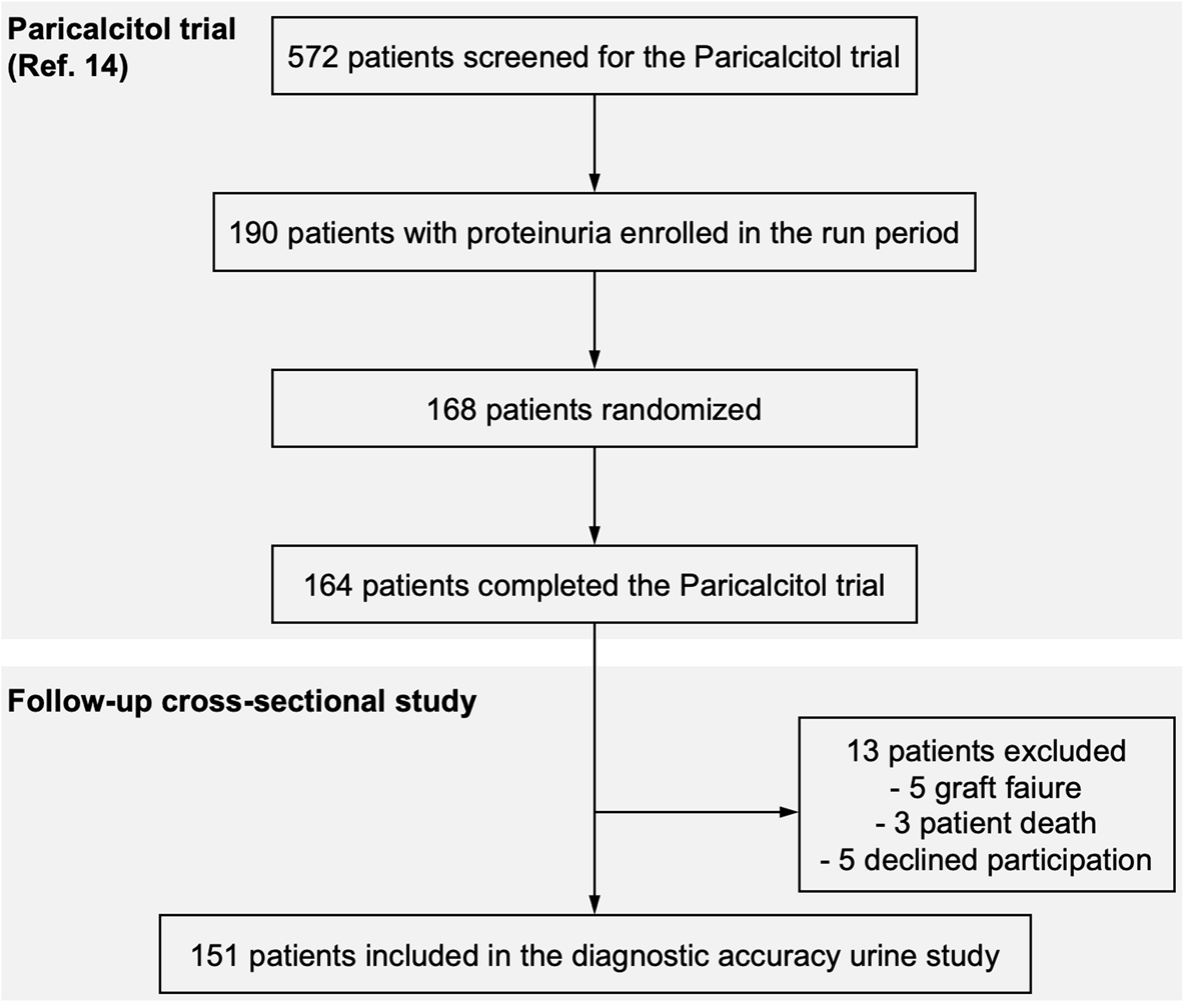
Flowchart showing the selection of study participants

From 190 patients that participated in the Paricalcitol trial, 168 patients were randomized, and 164 patients completed the treatment phase [14]. After Paricalcitol trial completion, 13 participants were excluded (5 graft failures and 3 patient deaths during follow-up, 5 patients declined to participate). This left a study group of 151 patients available for diagnostic urine analyses (Fig. 1). The baseline demographic, clinical, and laboratory characteristics of the study cohort are presented in Table 1.

**Table 1:** Baseline patient demographics, clinical and laboratory characteristics^*^

Variables	*N* = 151
***Demographic characteristics***	
Age (years)	56 ± 13
Male gender (%)	101 (67)
Body mass index (kg/m^2^)	25.6 ± 3.9
Original kidney disease	
diabetic glomerulosclerosis (%)	8 (5)
hypertension (%)	9 (6)
glomerulonephritis (%)	56 (37)
polycystic (%)	19 (13)
pyelonephritis/reflux (%)	14 (9)
other/undefined (%)	16 (11) / 29 (19)
***Clinical characteristics***	
Time post-transplant (years)	8.1 (3.1 to 12.9)
Prior indication allograft biopsy^a^ (%)	76 (50)
Histological diagnosis	
antibody-mediated rejection (%)	30 (40)
T-cell rejection (%)	22 (29)
recurrent glomerular disease (%)	7 (9)
other findings^b^ (%)	17 (22)
*De-novo* donor-specific antibodies^c^ (%)	28 (19)
Vital parameters	
systolic blood pressure (mmHg)	136 ± 17
diastolic blood pressure (mmHg)	76 ± 11
heart rate (beats/min)	74 ± 13
***Medication***	
ACEi/ARB (%)	138 (91)
diuretic	49 (32)
other antihypertensives	135 (89)
lipid-lowering treatments	96 (64)
glucose-lowering treatments	37 (25)
calcineurin inhibitor	151 (100)
mycophenolate	126 (83)
steroid	83 (55)
***Laboratory parameters***	
First morning urine collection	
protein-to-creatinine ratio (mg/mmol)	52 (31 to 124)
albumin-to-creatinine ratio (mg/mmol)	18 (6 to 76)
α-1 microglobulin-to-creatinine ratio (mg/mmol)	4.2 (2.3 to 6.8)
Second morning urine collection	
protein-to-creatinine ratio (mg/mmol)	52 (29 to 124)
albumin-to-creatinine ratio (mg/mmol)	22 (6 to 74)
α-1 microglobulin-to-creatinine ratio (mg/mmol)	4.1 (2.2 to 7.2)
24-h urine collection	
protein (mg/day/1.73m^2^)	490 (250 to 1160)
creatinine clearance (ml/min)	51 ± 23
Serum	
creatinine	127 ± 48
eGFR (ml/min/1.73m^2^)	53 ± 19
total cholesterol (mmol/L)	4.9 ± 1.1
LDL cholesterol (mmol/L)	2.8 ± 0.8
HDL cholesterol (mmol/L)	1.3 ± 0.5
triglycerides (mmol/L)	1.9 ± 1.2
calcium (mmol/L)	2.33 ± 0.14
phosphate (mmol/L)	0.98 ± 0.23
albumin	43 ± 3

^*^Data are presented as mean ± SD or median (interquartile range) for normally or skewed distributed data, respectively, or as total number (percentage)

^a^Data on prior allograft biopsies and histological diagnoses are based on Paricalcitol trial (Ref. [14])

^b^Include calcineurin inhibitor nephrotoxicity, hypertensive glomerulosclerosis, polyomavirus-associated nephropathy, and reflux nephropathy

^c^Determined at the time of most recent indication allograft biopsy

*Abbreviations*: *ACEi* angiotensin-converting enzyme inhibitor, *ARB* angiotensin receptor blocker, *eGFR* estimated glomerular filtration rate

Approximately 50% of patients had information on histologic phenotype of allograft injury before enrolment, and the most common histologic diagnosis was antibody-mediated rejection (AMR). Immunosuppressive regimens and other concomitant treatments are presented in Table 1. Overall, 138 patients (91%) received background angiotensin converting enzyme (ACE) inhibitor or angiotensin receptor blocker (ARB) in a fixed dose (Table 1).

### Performance of estimated urine protein excretion in first and second morning urine

**Fig. 2:**
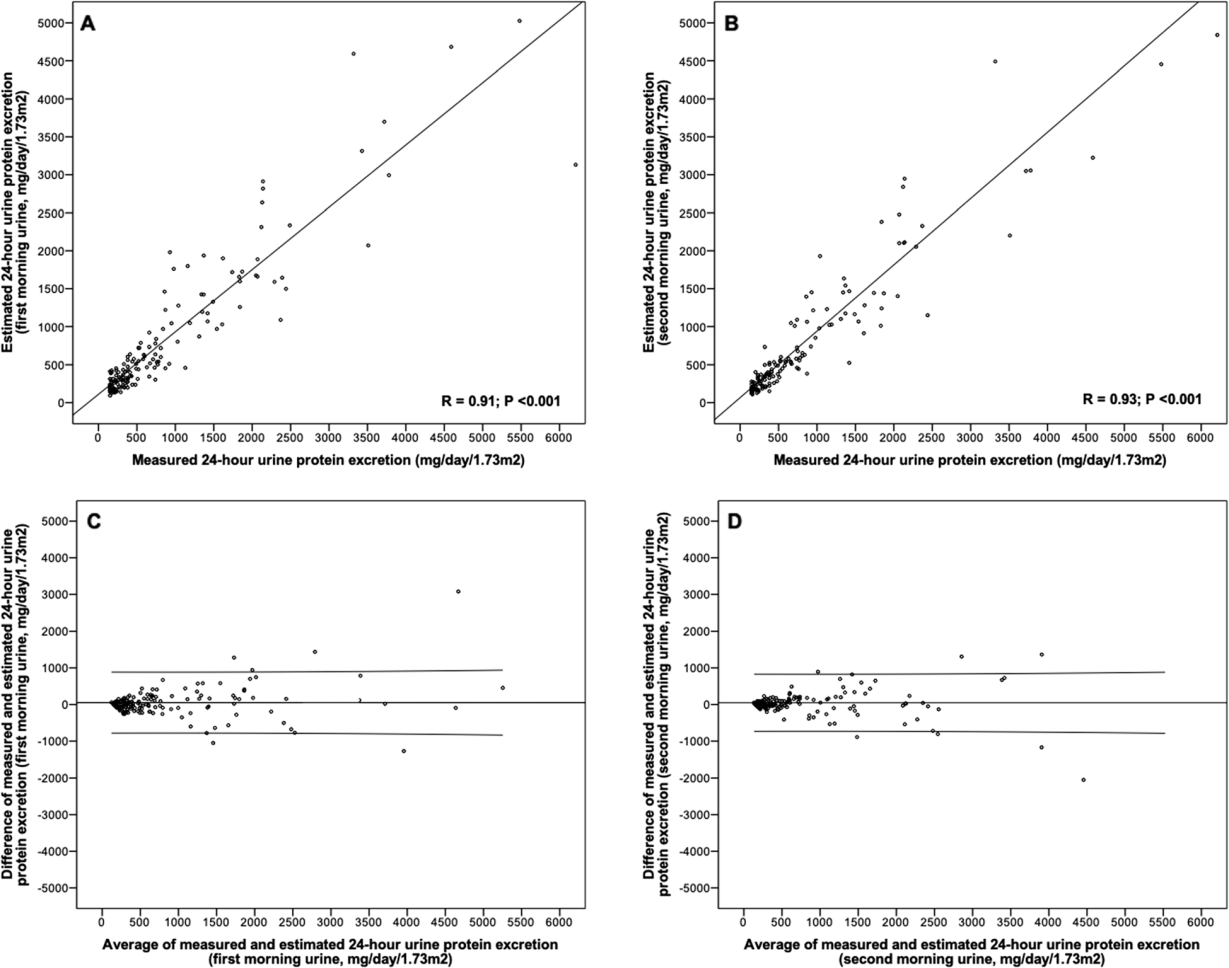
Scatter plot and Bland-Altman analysis comparing measured with estimated 24-h urine protein excretion for first (**a**, **c**) and second (**b**, **d**) morning spot urine collections. In Bland-Altman plots horizontal lines are drawn at the mean difference, and at the 95% limits of agreement (defined as the mean difference ± 1.96 times the standard deviation of the differences)

The performance of spot urine protein excretion in first and second morning urine collections as compared with 24-h proteinuria are presented in Table 2 and Fig. 2. The correlation coefficient between ePER in the first morning urine and 24-h proteinuria was 0.91 (95% CI 0.88 to 0.96; *R*^2^ = 0.83; *P* < 0.001; Fig. 2a), and between ePER in the second morning urine and 24-h proteinuria was 0.93 (95% CI 0.90 to 0.96; *R*^2^ = 0.86; *P* < 0.001; Fig. 2b). Bland-Altman analysis comparing measured and estimated 24-h urine protein excretion in first and second morning spot urine collections revealed higher variability after nephrotic range of proteinuria (> 3 g/day/1.73m^2^; Fig. 2c and d).

**Table 2:** Bias, precision, and accuracy of first and second morning spot urine estimated protein excretion compared with 24-h proteinuria

24-h proteinuria mean (SD) mg/day/1.73m^2^	n	Urine sample	Bias^a^ mg/day/1.73m^2^ (95% CI)	Percent bias^b^ % (95% CI)	Precision^c^ mg/day/1.73m^2^ (95% CI)	Accuracy^d^ within 15%, % (95% CI)	Accuracy^d^ within 30%, % (95% CI)	Accuracy^d^ within 50%, % (95% CI)
**150 to 299**	**45**	first morning	−46 (− 77 to − 16)	− 28 (− 44 to − 11)	102 (84 to 129)	22 (12 to 36)	56 (41 to 69)	71 (57 to 82)
201 (45)		second morning	−17 (− 37 to 3)*	−11 (− 21 to − 1)*	67 (55 to 85)*	36 (23 to 50)*	71 (57 to 82)*	89 (76 to 96)
Subgroup								
CrCl ≥60 ml/min	18	first morning	−6 (− 59 to 46)	−9 (− 37 to 19)	106 (80 to 159)	17 (5 to 40)	67 (44 to 84)	89 (66 to 98)
206 (42)		second morning	−5 (− 48 to 37)	−6 (− 26 to 15)	85 (64 to 127)	28 (12 to 51)	61 (39 to 79)	83 (60 to 95)
CrCl < 60 ml/min	26	first morning	−73 (− 109 to − 37)	−41 (− 61 to − 20)	91 (72 to 125)	26 (13 to 45)	48 (31 to 66)	59 (41 to 76)
198 (48)		second morning	−25 (−45 to − 4)**	−14 (− 25 to − 3)**	52 (41 to 71)**	41 (25 to 59)*	78 (59 to 89)*	93 (76 to 99)*
**300 to 999**	**65**	first morning	−2 (−64 to 59)	1 (− 8 to 10)	249 (212 to 301)	38 (28 to 51)	60 (48 to 71)	91 (81 to 96)
380 (54)		second morning	16 (−30 to 64)	2 (−6 to 10)	189 (161 to 229)	43 (32 to 55)	74 (62 to 83)	88 (77 to 94)
Subgroup								
CrCl ≥60 ml/min	23	first morning	75 (1 to 148)	13 (−1 to 27)	170 (131 to 241)	22 (9 to 42)	48 (29 to 67)	96 (77 to 100)
368 (54)		second morning	71 (5 to 137)	11 (−5 to 27)	152 (118 to 215)	39 (22 to 59)*	70 (49 to 85)*	87 (67 to 96)
CrCl < 60 ml/min	42	first morning	−44 (− 130 to 42)	−6 (− 17 to 5)	275 (226 to 351)	48 (33 to 62)	67 (51 to 79)	88 (75 to 95)
388 (54)		second morning	−12 (−76 to 51)	−2 (− 11 to 7)	202 (166 to 258)	45 (31 to 60)	76 (61 to 87)*	88 (75 to 95)
**≥1000**	**41**	first morning	245 (23 to 467)	9 (1 to 18)	703 (577 to 899)	44 (30 to 59)	73 (58 to 84)	98 (86 to 100)
2226 (1175)		second morning	168 (−54 to 390)	6 (−4 to 15)	704 (578 to 901)	42 (28 to 57)	78 (63 to 88)	93 (80 to 98)
Subgroup								
CrCl ≥60 ml/min	13	first morning	354 (− 283 to 990)	10 (−11 to 31)	1054 (756 to 1740)	31 (12 to 58)	62 (35 to 82)	100 (73 to 100)
2082 (1398)		second morning	312 (−89 to 712)	16 (−1 to 32)	663 (475 to 1094)	38 (18 to 65)	77 (49 to 93)	92 (65 to 100)
CrCl < 60 ml/min	28	first morning	195 (8 to 381)	9 (−1 to 18)	480 (380 to 653)	50 (33 to 68)	79 (60 to 90)	96 (81 to 100)
2292 (1078)		second morning	101 (− 180 to 381)	2 (−11 to 13)	720 (569 to 980)	43 (27 to 61)	79 (60 to 90)	93 (76 to 99)

^a^Bias was defined as the mean difference between the measured value of 24-h proteinuria and the estimated value from spot urine samples (measured-estimated)

^b^Percent bias was defined as (bias/24-h protein excretion) × 100

^c^Precision was defined as standard deviation of the mean bias

^d^Accuracy was defined as the percentage of estimated values within 15, 30, and 50% of the measured value

*P < 0.05; **P < 0.01

*Abbreviations*: *CrCl* creatinine clearance, *CI* confidence interval

In patients with low-grade proteinuria (150 to 299 mg/day/1.73m^2^), the ePER tended to overestimate the 24-h proteinuria, and the absolute overestimation was greater in first morning urine (Table 2). In contrast, in patients with moderate (300 to 999 mg/day/1.73m^2^) and high-grade proteinuria (≥1000 mg/day/1.73m^2^) the ePER tended to underestimate the 24-h proteinuria. The absolute underestimation was progressively grater as the degree of proteinuria increased and was generally lower in second morning urine and in patients with impaired kidney function. Differences in the absolute measures of agreement were not significantly different between first and second morning urine samples (Table 2). The percent bias ranged from 1 to 28% and was greater in patients with low-grade proteinuria (11 to 28%) than in patients with moderate and high-grade proteinuria (1 to 9%). The percent bias was similar in first and second morning urine, except in patients with low-grade proteinuria where it was significantly lower in the second morning urine (Table 2). The accuracies within 15 and 30% were modest (range 22 to 40% and 56 to 78%, respectively) and for the most part stronger in second than in first morning urine. The accuracy progressively increased with an increase in proteinuria and was better in patients with impaired kidney function (Table 2). For example, 56% of ePER in the first morning urine and 71% of ePER in the second morning urine would fall within 30% of the measure value for patients with mild proteinuria, as compared with 73 and 78% of ePER in the first and second morning urine, respectively, for patients with high-grade proteinuria. The accuracy within 50% was better and ranged from 71 to 98% in the first and 88 to 93% in the second morning urine collections (Table 2).

The performance of ePER to predict 24-h proteinuria was reanalyzed in a subgroup of study participants with information on histologic phenotype of allograft injury before enrolment (Table S1). In all histologic phenotypes, the absolute bias was once again greater with larger amount of proteinuria, and for the most part differences in the absolute measures of agreement were not significantly different between first and second morning urine collections. The relative agreement between ePER and 24-h proteinuria was stronger among patients with moderate and high-grade proteinuria, and there were no significant differences between first and second morning urine collections (Table S1). The accuracy within 15% was modest, but the accuracy within 30% and within 50% was stronger in all grades of proteinuria and different histologic phenotypes. Nevertheless, accuracy ranges were wide, probably relating to small number of patients within different histologic subgroups (Table S1).

### ROC curve analyses of proteinuria in first and second morning urine

The ROC curve analyses (Table 3) show the fraction of true positive results (sensitivity) and false positive results (1 – specificity) for various cutoff levels of 24-h proteinuria. In general, the PCR (ePER) in first and in second morning urine demonstrated good discriminatory ability. For example, the PCR (ePER) threshold that gave the maximal sensitivity and specificity for abnormal amounts of protein in the urine (i.e., > 150 mg/day) was 27 mg/mmol (235 mg/day/1.73m^2^) in the first morning urine, and 26 mg/mmol (225 mg/day/1.73m^2^) in the second morning urine; at this threshold, the sensitivity was 83% and the specificity was 86% (Table 3). The sensitivity and specificity both increased with increasing amounts of proteinuria. The test performance of PCR (ePER) in the first morning urine was similar to the PCR (ePER) in the second morning urine (Table 3).

**Table 3:** Receiver operator characteristic (ROC) analysis of proteinuria in first and second morning spot urine collections

24-h proteinuria (mg/day/1.73m^2^)	Urine sample	PCR / ePER cutoff point (mg/mmol / mg/day/1.73m^2^)	Area under the ROC curve (95% CI)	Sensitivity of PCR/ePER % (95% CI)	Specificity of PCR/ePER % (95% CI)
**> 150**	first morning	27 / 235	0.89 (0.82 to 0.96)	83 (74 to 89)	86 (77 to 92)
	second morning	26 / 225	0.92 (0.86 to 0.98)	83 (74 to 89)	86 (77 to 92)
**> 300**	first morning	39 / 345	0.92 (0.88 to 0.96)	84 (75 to 90)	86 (77 to 92)
	second morning	38 / 335	0.96 (0.93 to 0.99)	88 (80 to 93)	89 (81 to 94)
**> 1000**	first morning	107 / 940	0.98 (0.97 to 0.99)	93 (83 to 98)	96 (89 to 99)
	second morning	105 / 925	0.98 (0.97 to 0.99)	89 (81 to 94)	96 (89 to 99)

*Abbreviations*: *PCR* protein-to-creatinine ratio, *ePER* estimated protein excretion rate, *CI* confidence interval

Whereas the presence or absence of allograft injury is known with a high degree of certainty in patients who have undergone kidney biopsy, the discriminatory ability of PCR (ePER) for different cutoff levels of 24-h proteinuria was tested using only the results from the patients with allograft injury demonstrated in prior indication kidney biopsies. This evaluation revealed that PCR (ePER) of 20 mg/mmol (175 mg/day/1.73m^2^) in the first and second morning urine had a sensitivity of 96% and a specificity of 100% for the diagnosis of proteinuria > 150 mg/day. The sensitivity and specificity both remained high with an increase in proteinuria and the test performance of PCR (ePER) being similar in the first and second morning urine (Table S2).

### Association of spot urine protein excretion with 24-h proteinuria

Urinary levels of PCR, ACR, and α-1 MCR were significantly associated with the levels of 24-h proteinuria, although this association was less pronounced with α-1 MCR. All associations were comparable in the first and second morning spot urine collections (Table 4).

**Table 4:** Levels of spot urine protein, albumin, and α-1 microglobulin excretion according to 24-h proteinuria^*^

Parameter	24-hour proteinuria (mg/day/1.73m^**2**^)			***P*** value^a^
	150–299	300–999	≥ 1000	
**PCR (mg/mmol)**				
first morning urine	24 (19 to 36)	57 (37 to 72)	189 (139 to 282)	< 0.001
second morning urine	22 (19 to 29)	56 (42 to 68)	188 (133 to 290)	< 0.001
**ACR (mg/mmol)**				
first morning urine	5 (2 to 13)	17 (10 to 34)	143 (77 to 193)	< 0.001
second morning urine	5 (2 to 8)	22 (12 to 34)	123 (83 to 216)	< 0.001
**α-1 MCR (mg/mmol)**				
first morning urine	3.2 (1.6 to 5.5)	5.4 (3.0 to 7.8)	4.3 (3.1 to 6.0)	0.002
second morning urine	3.4 (1.8 to 4.5)	5.9 (2.5 to 8.4)	4.8 (2.8 to 8.1)	0.009

^*^Data are presented as median (interquartile range)

^a^Differences were tested by the Kruskal–Wallis test for non–normally distributed data

*Abbreviations*: *ACR* albumin-to-creatinine ratio, *α-1 MCR* α-1 microglobulin-to-creatinine ratio, *PCR* protein-to-creatinine ratio

### Association of spot urine protein excretion with allograft histology

**Fig. 3:**
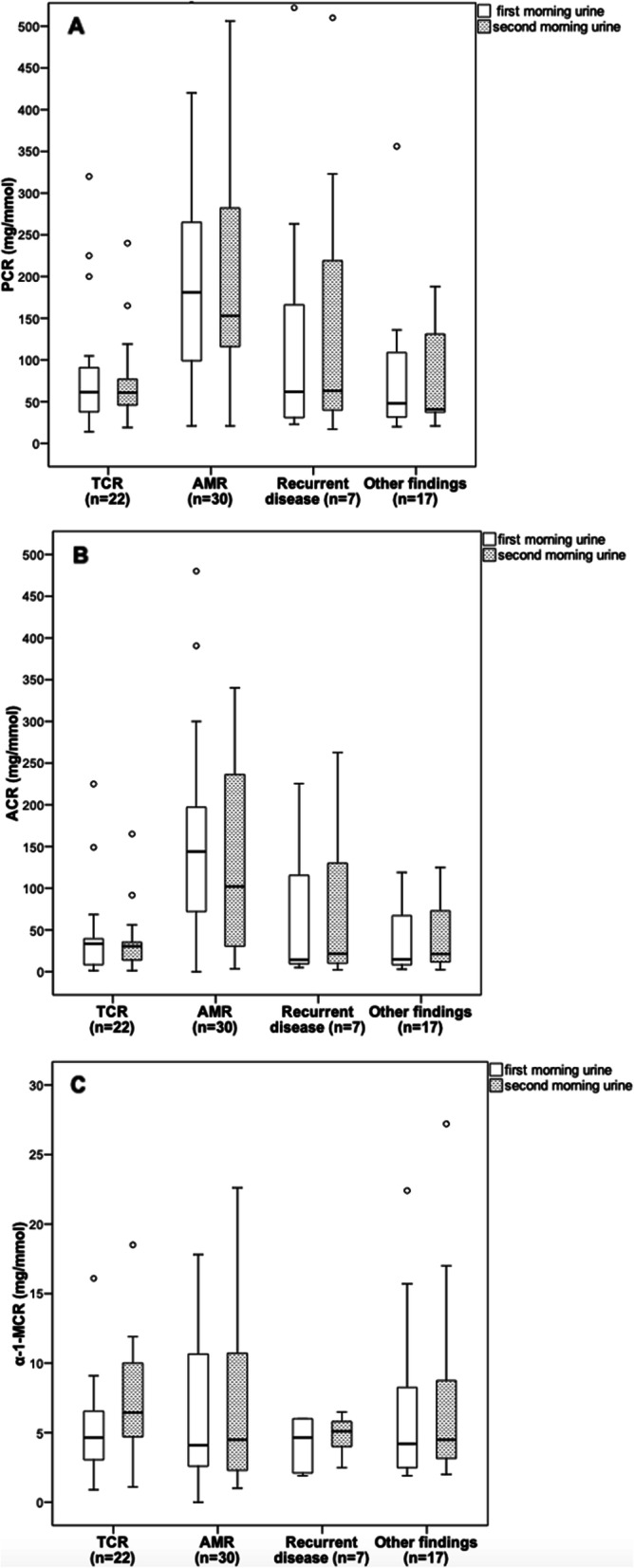
Box-and-whisker plots show PCR (**a**), ACR (**b**), and α-1 MCR (**c**) in first and second morning spot urine matched to 76 patients with information on histologic phenotype of allograft injury before enrolment in the study. The horizontal line within each box represents the median, the bottom and top of each box represent the 25th and 75th percentile values, the I bars represent the 10th and 90th percentile values, and circles indicate outliers. PCR, protein-to-creatinine ratio; ACR, albumin-to-creatinine ratio; α-1 MCR, α-1 microglobulin-to-creatinine ratio; TCR, T-cell rejection; AMR, antibody mediated rejection; other histologic findings include calcineurin inhibitor nephrotoxicity, hypertensive glomerulosclerosis, polyomavirus-associated nephropathy, and reflux nephropathy

Figure 3 displays levels of urine protein, albumin, and α-1 microglobulin in first and second morning spot urine collections classified according to allograft histology in the subgroup of patients with information on the histologic phenotype of allograft injury before enrolment. PCR and ACR levels differed significantly across different histologic phenotypes of allograft injury (*P* < 0.001) and were highest in patients with AMR. In contrast, levels of α-1 MCR did not significantly differentiate between various histologic phenotypes (*P* = 0.984 and *P* = 0.461 for the first and second morning urine, respectively). Pairwise group comparisons showed no significant differences between the levels of PCR, ACR, and α-1 MCR in the first and second morning urine across histologic phenotypes (Fig. 3).

## Discussion

To our knowledge, this study provides the first assessment of bias, precision and accuracy of ePER determined from PCR in the first and second morning spot urine collections in kidney transplant recipients. Our data showed excellent correlation and uniform agreement below nephrotic-range proteinuria, and moderate bias, precision, and accuracy of ePER in both the first and the second morning spot urine collections at predicting 24-h proteinuria. Furthermore, measures of agreement improved with an increase in proteinuria. Differences in the measures of agreement were not significantly different between first and second morning urine, except in low-grade proteinuria where the performance of ePER in the second morning urine was slightly better. This finding may have clinical utility given the fact that analysis of the second morning urine sample is more convenient in the outpatient settings. Finally, measures of agreement between ePER in the first and second morning urine and 24-h proteinuria were similar across different histologic phenotypes of allograft injury.

Proteinuria has been associated with progressive kidney disease, graft loss and mortality in kidney transplant recipients [1–3]. There have been several other studies linking proteinuria with allograft failure and death [22–24]. In these analyses, the average adjusted relative risk for allograft failure for patients with proteinuria was 2.7, and the average adjusted relative risk of death was 1.98 [25]. Moreover, posttransplant proteinuria is highly specific for transplant glomerulopathy, microcirculation inflammation, and de novo/recurrent glomerular disease and the prognosis of these specific disease processes is primarily determined by the associated degree of proteinuria [26]. Thus, accurate assessment of proteinuria is necessary for prognostic as well as diagnostic purposes and may be a target for therapy.

Spot sample urine measurements have become a standard of care for the assessment of 24-h proteinuria in kidney transplant recipients and KDIGO guidelines recommend using the PCR as an alternate to the 24-h collection method [27]. However, the validity of spot urine protein measurement in this population remains unclear. Most diagnostic accuracy studies evaluating data on PCR only reported correlation with 24-h proteinuria, while several studies also reported on sensitivity and specificity of PCR using various cutoff values [8]. High correlation does not imply good agreement between two methods of measurement, because it evaluates only the linear association of two sets of observations. The diagnostic accuracy studies have also examined the sensitivity and specificity of PCR in relation to 24-h proteinuria. Both are statistical measures of the performance of a binary classification and as such, none of these measures gives the clinician information about quantitative accuracy of the test. To date, only one study evaluated bias, precision and accuracy of PCR measurements and 24-h proteinuria in kidney transplant recipients [12]. However, the authors did not provide information which urine sample was analyzed and weather spot urine and 24-h urine were collected on the same day. This may have contributed to the suboptimal agreement between PCR and 24-h protein excretion as previous studies had demonstrated that random spot urine protein measurements show poor correlation and poor agreement with 24-h collections [13, 28, 29].

In our study, we used first and second morning void urine collections. It must be emphasized that, although these are spot urine collections, they are not random collections, because they are the first or second voids of the day. Previous data in CKD patients suggested that consistency in the timing of collections may improve the agreement between spot PCR measurements and 24-h urine collections [29, 30]. Our data demonstrated excellent correlation between estimated and measured 24-h proteinuria with sensitivity and specificity values 83% or greater, depending on urine sample and cutoff used. The sensitivity and specificity both increased with greater proteinuria and were similar for the first and second morning urine collections. For example, the optimal cutoff for PCR in the first morning urine was 27 mg/mmol for 24-h proteinuria > 150 mg/day and 26 mg/mmol in the second morning urine. At these cutoff levels the sensitivity was 83%, and the specificity was 86% for the diagnosis of proteinuria in the first and second morning urine. For diagnosing high-grade proteinuria > 1 g/day, the optimal cutoff values were 107 (sensitivity 93%, specificity 96%) and 105 mg/mmol (sensitivity 89%, specificity 96%) in the first and second morning urine, respectively. These sensitivity and specificity data are consistent with earlier reports [8]. The discriminatory ability of PCR in first and second morning urine for different cutoff levels of 24-h proteinuria remained similar in patients with allograft injury demonstrated in the preceding indication kidney biopsies.

Like for the estimation of GFR, one should know the absolute measures of agreement between ePER and 24-h proteinuria (i.e., bias, precision and accuracy). Accuracy represents the most useful analysis for the clinician, since it takes into account both bias and precision by expressing how many estimates are dispersed within a given range of their respective measurements [31]. Because day-to-day fluctuations in proteinuria have been reported to be as high as 37% [32], accuracy within 30% best provides the proportion of estimates not deviating from measured 24-h protein excretion. In our study the accuracies within 30% ranged from 56 to 78% and were slightly better than those reported in the study of Akbary et al. (47 to 56%) [12]. No significant differences in absolute measures of agreement between first and second morning urine collections were found, except in low-grade proteinuria where the performance of ePER in the second morning urine was better. In this regard, the second morning spot urine may be particularly relevant, because it is easier to collect, and probably represents as uniform and achievable way as possible to collect urine among outpatients. In addition, performance increased with an increase in proteinuria and was better in patients with decreased allograft function. This finding is important given the fact that major diagnostic (e.g., biopsy) or therapeutic (e.g., change in immunosuppression) decisions are most commonly indicated in patients with a decrease in kidney function, new-onset or worsening proteinuria.

Previous diagnostic accuracy studies did not provide information on allograft histology and on the type of urine protein excretion associated with different levels of proteinuria, which may influence the accuracy of PCR measurements. With an increase in proteinuria, urinary levels of individual proteins albumin and α-1 microglobulin were also increased. Nevertheless, α-1 microglobulin increased in parallel with albumin excretion only in patients with low to moderate proteinuria (i.e. < 1 g/day), while high-grade proteinuria was mainly associated with an increase in albumin excretion. This is in line with previous observations, which showed that low-grade proteinuria and small increases in urinary albumin may result from proximal tubular damage where urinary albumin often increases in parallel with α-1 microglobulin [33]. In those patients with marked glomerular pathology heavy proteinuria composed overwhelmingly of albumin is common, and thus the correlation of total urinary protein and albumin with lower molecular weight tubular proteins may be lost. In our study, the predominance of albuminuria and relatively lower amounts of tubular proteins may explain greater diagnostic accuracy of ePER in patients with high-grade proteinuria. Spot urine protein and albumin excretion were greater in patients with previous diagnosis of AMR and recurrent glomerular disease than in patients with T-cell rejection or other non-rejection findings. In contrast, α-1 microglobulin excretion was not significantly associated with different histological phenotypes. These associations were similar for the first and second morning urine samples. However, this study did not examine whether spot urine protein or albumin excretion could predict specific histologic phenotypes of allograft injury. In previous studies, urine protein profiles alone have not predicted specific histologic injury phenotypes [33, 34].

This study has some limitations that should be acknowledged. First, our study only included a Caucasian population and a deceased donor kidney source. This may limit external validity to other more diverse patient populations with a higher proportion of living donor allografts and non-Caucasians. Second, number of patients with nephrotic-range proteinuria was low and correlations between estimated and measured values were more consistent for urines with proteinuria below 3 g/day. Therefore, 24-h urine collection should still be needed for proteinuria quantification in patients with severe proteinuria. Third, surveillance biopsies were not performed and only a small number of patients with histologic data on preceding biopsies were included, making it difficult to comment on the performance of the ePER in association with different phenotypes of allograft injury. Finally, we do not have outcome data to determine which measure of proteinuria (i.e., ePER in the first or second morning urine sample, or 24-h collection) is most strongly associated with transplant outcomes.

## Conclusions

In conclusion, commonly available ePER determined from PCR in the first and second morning urine allow estimation of an individual’s 24-h protein excretion with excellent correlation and uniform agreement below nephrotic-range proteinuria, and with moderate bias, precision, and accuracy. Better diagnostic performance of ePER measurements in recipients with greater proteinuria may prove useful in patients with allograft dysfunction and injury. Given the similar accuracy of spot urine protein measurements in the first and second morning urine, second morning spot collection can be used for monitoring protein excretion in the outpatients.

## Supplementary Information

**Additional file 1: Supplementary file 1.** Patients and Methods. Details on the study measurements. **Table S1.** Bias, precision, and accuracy of first and second morning spot urine protein excretion compared with 24-h proteinuria in 76 patients with information on histologic phenotype of allograft injury before enrolment in the study. **Table S2.** Receiver operator characteristic (ROC) analysis of proteinuria in first and second morning spot urine collections in 76 patients with information on histologic phenotype of allograft injury before enrolment in the study.

## Data Availability

The datasets generated during and/or analyzed during the current study are available from the corresponding author on reasonable request.

## Abbreviations

ACE: Angiotensin converting enzyme
ACR: Albumin-to-creatinine ratio
AMR: Antibody-mediated rejection
ARB: Angiotensin receptor blocker
CI: Confidence interval
CrCl: Creatinine clearance
eGFR: Estimated glomerular filtration rate
HLA: Human leukocyte antibody
α-1 MCR: α-1 microglobulin-to-creatinine ratio
ePER: Estimated protein excretion rate
PCR: Protein-to-creatinine ratio
ROC: Receiver operator characteristic
SD: Standard deviation
TCR: T-cell rejection
