# Supplementary material for: First and second morning spot urine protein measurements for the assessment of proteinuria: a diagnostic accuracy study in kidney transplant recipients
Source: BMC Nephrol. 2021 May 22;22:192. doi: 10.1186/s12882-021-02406-x (PMC8141254; doi:10.1186/s12882-021-02406-x)
Supplement: Supplementary file 1 — Additional file 1: Supplementary file 1. Patients and Methods. Details on the study measurements. Table S1. Bias, precision, and accuracy of first and second morning spot urine protein excretion compared with 24-h proteinuria in 76 patients with information on histologic phenotype of allograft injury before enrolment in the study. Table S2. Receiver operator characteristic (ROC) analysis of proteinuria in first and second morning spot urine collections in 76 patients with information on histologic phenotype of allograft injury before enrolment in the study. [file 12882_2021_2406_MOESM1_ESM.docx]

**SUPPLEMENTARY DOCUMENTATION**

**First and second morning spot urine protein measurements for the assessment of proteinuria: a diagnostic accuracy study in kidney transplant recipients**

Maja Mrevlje^1,2^, Manca Oblak^1^, Gregor Mlinšek^1^, Jelka Lindič^1^, Jadranka-Buturović-Ponikvar^1,3^, and Miha Arnol^1,3*^

^1^Department of Nephrology, University Medical Centre Ljubljana, Slovenia

^2^General Hospital Izola, Department of internal medicine, Izola, Slovenia

^3^Faculty of Medicine, University of Ljubljana, Slovenia

**Supplement 1. Patients and Methods. Details on the study measurements**

For each study participant, 24-hour urine was collected for total protein excretion and creatinine clearance measurement, first and second morning urine samples were collected for protein-to-creatinine ratio, albumin-to-creatinine ratio, and α-1 microglobulin-to-creatinine ratio measurements, and blood sample was taken after overnight fasting. Blood pressure was evaluated at 1-minute intervals by an automatic device with the patient in a semisupine position. The mean of three readings was recorded. Patients were weighed and had their height measured. Body surface area was estimated by the DuBois formula (1).

Urine and serum creatinine were measured using non-isotope-dilution mass spectrometry standardized modified Jaffe reaction (calibration traceable to IDMS). Spot and 24-hour urine protein were measured by pyrogallol red-molybdate complex formation using a timed endpoint method. Measurements were performed on Dimension Xpand Plus Integrated Chemistry System using manufacturer’s reagents (Siemens Healthcare GmbH, Erlangen, Germany). Urine albumin and alpha-1-microglobuline were measured by means of immunonephelometry using BN ProSpec System for urine protein analysis (Siemens Healthcare GmbH, Erlangen, Germany). Blood electrolytes and lipids were determined by using an automated multianalyzer (ADVIA 1800 Chemistry System; Siemens Healthcare GmbH, Erlangen, Germany).

Creatinine clearance was calculated from creatinine concentrations in serum and 24-hour urine collections, and eGFR was calculated using the four-variable Modification of Diet in Renal Disease (MDRD) formula, given in International System units: 186 × (serum creatinine/88.4)^−1.154^ × age^−0.203^ × 0.742 if female x 1.210 if black (2).

1. DuBois D, DuBois E. A formula to estimate the approximate surface area if height and weight be known. Arch Intern Med 1916; 17: 863-71.

2. Levey AS, Greene T, Kusek JW, Beck GJ. A simplified equation to predict glomerular filtration rate from serum creatinine. J Am Soc Nephrol 2000; 11: A0828.

**Table S1.** Bias, precision, and accuracy of first and second morning spot urine protein excretion compared with 24-hour proteinuria in 76 patients with information on histologic phenotype of allograft injury before enrolment in the study.

| **24-hour proteinuria** mean (SD) mg/day/1.73m^2^ | **n** | **Urine sample** | **Bias**^a^ mg/day/1.73m^2^ (95% CI) | **Percent bias**^b^ % (95% CI) | **Precision**^c^ mg/day/1.73m^2^ (95% CI) | **Accuracy**^d^ within 15%, % (95 % CI) | **Accuaracy**^d^ within 30%, % (95% CI) | **Accuracy**^d^ within 50%, % (95% CI) |
| --- | --- | --- | --- | --- | --- | --- | --- | --- |
| **150 to 299**  189 (44)  Subgroup | **8** | first morning  second morning | -17 (-66 to 33)  -38 (-82 to 5) | -13 (-42 to -16)  -22 (-44 to 1) | 59 (39 to 120)  52 (34 to 105) | 25 (6 to 60)  50 (22 to 78) | 75 (40 to 93)  75 (40 to 93) | 75 (40 to 93)  75 (40 to 93) |
| TCR  160 (14)  AMR  240 (42)  Recurrent disease  205 (35)  Other findings^e^  175 (25) | 2  2  2  2 | first morning  second morning  first morning  second morning  first morning  second morning  first morning  second morning | 10 (-177 to 179)  -31 (-264 to 204)  45 (-152 to 241)  9 (-226 to 244)  -25 (-212 to 163)  -70 (271 to 229)  -88 (-120 to -56)  -62 (-249 to 126) | -2 (-164 to 161)  -19 (-150 to 92)  18 (-35 to 71)  4 (-95 to 110)  -10 (-83 to 62)  -31 (-110 to 73)  -48 (-69 to -27)  -41 (-117 to 77) | 64 (29 to 614)  26 (14 to 414)  22 (10 to 351)  26 (14 to 414)  43 (22 to 486)  49 (25 to 581)  14 (7 to 223)  23 (11 to 366) | 0 (0 to 71)  50 (10 to 90)  50 (10 to 90)  100 (29 to 100)  50 (10 to 90)  50 (10 to 90)  0 (0 to 71)  0 (0 to 71) | 100 (29 to 100)  100 (29 to 100)  100 (29 to 100)  100 (29 to 100)  100 (29 to 100)  50 (10 to 90)  0 (0 to 71)  50 (10 to 90) | 100 (29 to 100)  100 (29 to 100)  100 (29 to 100)  100 (29 to 100)  100 (29 to 100)  50 (10 to 90)  50 (10 to 90)  50 (10 to 90) |
| **300 to 999**  574 (208)  Subgroup | **36** | first morning  second morning | -20 (-119 to 79)  8 (-61 to 77) | 2 (-11 to 15)  2 (-8 to 12) | 294 (238 to 384)  205 (166 to 267) | 39 (25 to 55)  53 (37 to 68)* | 64 (48 to 78)  78 (62 to 89) | 89 (74 to 96)  86 (71 to 94) |
| TCR  583 (214)  AMR  690 (190)  Recurrent disease  503 (267)  Other findings^e^  501 (181) | 18  6  3  9 | first morning  second morning  first morning  second morning  first morning  second morning  first morning  second morning | -74 (-248 to 100)  -1 (-98 to 97)  63 (-204 to 330)  84 (-155 to 324)  33 (-137 to 202)  65 (-150 to 279)  17 (-171 to 205)  -46 (-230 to 139) | -6 (-29 to 16)  -1 (-14 to 13)  10 (-22 to 42)  11 (-17 to 39)  5 (-30 to 40)  10 (-23 to 42)  11 (-16 to 38)  -1 (-31 to 28) | 351 (263 to 526)  197 (148 to 295)  254 (159 to 623)  228 (142 to 559)  68 (39 to 300)  86 (50 to 380)  244 (165 to 467)  240 (162 to 460) | 28 (12 to 51)  56 (34 to 75)*  33 (9 to 70)  50 (19 to 81)  100 (38 to 100)  67 (20 to 94)  44 (19 to 73)  44 (19 to 73) | 56 (34 to 75)  83 (60 to 95)*  67 (30 to 91)  83 (42 to 99)  100 (38 to 100)  100 (38 to 100)  67 (35 to 88)  56 (27 to 81) | 83 (60 to 95)  89 (66 to 98)  100 (56 to 100)  83 (42 to 99)  100 (38 to 100)  100 (38 to 100)  89 (54 to 100)  78 (44 to 95) |
| **≥1000**  2392 (1267)  Subgroup  TCR  1585 (785)  AMR  2365 (1138)  Recurrent disease  3780 (3436)  Other findings^e^  2300 (991) | **32**  2  22  2  6 | first morning  second morning  first morning  second morning  first morning  second morning  first morning  second morning  first morning  second morning | 224 (-55 to 503)  84 (-183 to 350)  -223 (-985 to 540)  40 (-112 to 193)  166 (-54 to 386)  140 (-172 to 452)  1617 (-1695 to 2018)  543 (-997 to 1106)  121 (-853 to 1096)  260 (-676 to 1198) | 6 (-4 to 16)  1 (-10 to 11)  -5 (-147 to 138)  3 (-21 to 27)  6 (-6 to 17)  6 (-5 to 17)  30 (-211 to 272)  1 (-273 to 275)  3 (-30 to 36)  20 (-23 to 64) | 774 (621 to 1029)  740 (593 to 983)  641 (327 to 2045)  40 (20 to 437)  496 (382 to 709)  704 (541 to 948)  2066 (921 to 3295)  1170 (596 to 3733)  928 (580 to 2276)  893 (557 to 2190) | 44 (28 to 61)  44 (28 to 61)  0 (0 to 71)  100 (29 to 100)  50 (31 to 69)  45 (27 to 65)  50 (10 to 90)  100 (29 to 100)  33 (9 to 70)  33 (9 to 70) | 75 (58 to 87)  81 (64 to 91)  100 (29 to 100)  100 (29 to 100)  77 (56 to 90)  86 (66 to 96)  50 (10 to 90)  100 (29 to 100)  67 (30 to 91)  50 (19 to 81) | 100 (87 to 100)  94 (78 to 99)  100 (29 to 100)  100 (29 to 100)  100 (82 to 100)  95 (76 to 100)  100 (29 to 100)  100 (29 to 100)  100 (56 to 100)  83 (42 to 99) |

^a^Bias was defined as the mean difference between the measured value of 24-hour proteinuria and the estimated value from spot urine samples (measured-estimated).

^b^Percent bias was defined as (bias/24-hour protein excretion) x 100.

^c^Precision was defined as standard deviation of the mean bias.

^d^Accuracy was defined as the percentage of estimated values within 15%, 30%, and 50% of the measured value.

^e^Include calcineurin inhibitor nephrotoxicity, hypertensive glomerulosclerosis, polyomavirus-associated nephropathy, and reflux nephropathy.

**P*<0.05

Abbreviations: AMR, antibody-mediated rejection; CI, confidence interval; PCR, protein-to-creatinine ratio; TCR, T-cell rejection.

**Table S2.** Receiver operator characteristic (ROC) analysis of proteinuria in first and second morning spot urine collections in 76 patients with information on histologic phenotype of allograft injury before enrolment in the study.

| **24-hour proteinuria** (mg/day/1.73m^2^) | **Urine sample** | **PCR / ePER cut point** (mg/mmol / mg/day/1.73m^2^) | **Area under the ROC curve** (95% CI) | **Sensitivity of PCR / ePER** % (95% CI) | **Specificity of PCR / ePER** % (95% CI) |
| --- | --- | --- | --- | --- | --- |
| **>150** | first morning  second morning | 20 / 175  20 / 175 | 0.96 (0.92 to 0.99)  0.96 (0.92 to 0.99) | 96 (89 to 99)  96 (89 to 99) | 100 (95 to 100)  100 (95 to 100) |
| **>300** | first morning | 43 / 375 | 0.98 (0.96 to 1.0) | 97 (91 to 99) | 88 (80 to 93) |
|  | second morning | 38 / 335 | 0.96 (0.91 to 0.99) | 94 (87 to 97) | 80 (71 to 87) |
| **>1000** | first morning | 109 / 955 | 0.98 (0.96 to 1.0) | 91 (84 to 96) | 93 (86 to 97) |
|  | second morning | 102 / 900 | 0.98 (0.96 to 1.0) | 89 (81 to 94) | 95 (89 to 98) |

Abbreviations: PCR, protein-to-creatinine ratio; ePER, estimated protein excretion rate; CI, confidence interval.
